# Dosimetric analysis of half-field-based VMAT with the deep inspiration breath-hold technique for left breast cancer patients following breast-conserving surgery

**DOI:** 10.3389/fonc.2024.1418723

**Published:** 2024-11-21

**Authors:** Weiwei Wu, Hui Yin, Zhiwei Liu, Lipeng Liu, Chengjian Xiao, Ying Xiao, Jinquan Ding, Qungui Zhang, Hailiang Guo

**Affiliations:** ^1^ Center of Radiation Oncology, Ganzhou Cancer Hospital, Ganzhou, Jiangxi, China; ^2^ Medical Imaging Department, Ganzhou Cancer Hospital, Ganzhou, Jiangxi, China; ^3^ Department of Oncology, the First Affiliated Hospital of Gannan Medical University, Ganzhou, Jiangxi, China

**Keywords:** deep inspiration breath-holding, half beam, VMAT, breast cancer, dosimetry

## Abstract

**Objective:**

Compared the dosimetric characteristics of half-field-based VMAT and half-field-based IMRT for left breast cancer patients combined with deep inspiration breath-hold (DIBH) and free breathing (FB) techniques.

**Methods:**

Twenty-one left breast cancer patients were included. Each patient underwent DIBH and FB CT scans, IMRT and VMAT plans in half-field beam mode for both breathing techniques, resulting in four plans: FB-IMRT (F-IMRT), FB-VMAT (F-VMAT), DIBH-IMRT (D-IMRT) and DIBH-VMAT (D-VMAT). The conformity index (CI), homogeneity index (HI), and the doses received at the heart, left anterior descending (LAD), left lung, right breast, and right lung, were compared among plans.The correlation between the difference in the volume of lung_L (ΔLVL) and the difference in the mean dose (ΔDmean) of lung_L under the DIBH and FB plans, the correlation between the difference in the heart-chest distance (ΔHCD) and the ΔDmean of the heart,LAD under the DIBH and FB plans.

**Results:**

The D-VMAT plan lower lung_L V5 than both the F-IMRT and F-VMAT plans (*p*<0.05), The D-VMAT plan lower values for V10, V20, V30, and Dmean than did the other plans (*p* < 0.05). For the heart, the D-VMAT plan lower V5, V10, V20, and Dmean values than did the other plans (*p* < 0.05). The D1% and Dmax of the heart and the Dmax and Dmean of the LAD obtained with the D-VMAT plan were lower than those obtained with the F-IMRT and F-VMAT plans (*p* < 0.05). ΔHCD exhibited correlation with the ΔDmean of the LAD between the D-VMAT and F-IMRT plans and between the D-VMAT and F-VMAT plans (R = -0.765 and -0.774, respectively, *p* = 0.000).

**Conclusion:**

the D-VMAT plan offered enhanced protection for OARs. The integration of the DIBH technique with half-field and VMAT technology in the D-VMAT plan offers a superior dose distribution.

## 1 Introduction

Breast cancer is currently one of the most common malignancies in women, and its incidence has been increasing in recent years (1). Adjuvant radiotherapy following breast-conserving surgery can reduce the risk of local recurrence and distant metastasis as well as improve the overall survival rate of patients with early breast cancer (2). Postoperative radiotherapy for breast cancer can cause radiation damage to the heart, lung and mammary glands of the healthy side adjacent to the target area, leading to an increased risk of heart-related adverse events such as ischemic heart disease, valve disease, arrhythmia, congestive heart failure and secondary primary lung and breast cancer (3, 4). Among them, heart-related adverse events have become the primary threat to the long-term survival of patients with early breast cancer (5). In particular, radioactive cardiac injury (also called radiation-induced heart disease, or RIHD) in is a serious late complication of radiotherapy for breast cancer (6). The probability of a patient developing RIHD is positively correlated with the mean cardiac dose (or mean heart dose, MHD); specifically, for every 1 Gy increase in the MHD received by the patient, the probability of developing ischemic heart disease (IHD) increases by approximately 7.4% (7), and no minimum dose threshold has been identified (8). How can the dose delivered to breast cancer patients, and therefore the probability of radiation-related complications, be reduced? To answer this question, various technologies are constantly being developed and applied in radiotherapy for breast cancer treatment.

The deep inspiration breath-hold (DIBH) technique is one of the most popular methods used in breast cancer radiotherapy. A DIBH is achieved through voluntary deep inhalation to a certain limit during radiotherapy; this causes the chest and lung volume to expand and the heart to move to the lower right. This technique increases the distance between the heart and the chest wall while ensuring coverage of the best target area and reduces the irradiation density of lung tissue, which can effectively protect important organs, especially the heart and major blood vessels, during radiotherapy (9–11). Moreover, this technique has good repetition and stability (12, 13) and is routinely recommended for radiotherapy of the left breast.

Following breast-conserving surgery, breast cancer patients commonly undergo radiotherapy via techniques such as three-dimensional conformal radiotherapy (3DCRT), intensity-modulated radiation therapy (IMRT), and volumetric modulated arc therapy (VMAT) to minimize radiation exposure to critical organs (14). VMAT is an advanced mode of IMRT, in which the beam intensity is adjusted during continuous rotation of the gantry. VMAT technology typically has greater overall performance than other radiotherapy techniques used in postbreast cancer surgery radiotherapy (15–17). Nevertheless, in comparison to tangential IMRT, VMAT has a greater low-dose range (18–20), which can be mitigated with a suitable field layout (21). The half-beam field is a special form of asymmetric field in which one side of the tungsten gate is pushed to the central axis and fixed; consequently, the corresponding side is referred to as the half-beam field, while the other side is composed of a multileaf collimator (MLC). Li et al. (22) found that the field scattering dose of the half-beam field was significantly lower than that of a symmetrical field. The application of fixed half-beam technology to radiotherapy for breast cancer can limit the radiation dose range, reduce the influence of scattering radiation on lung tissue, and obtain a better dose distribution in radiotherapy for breast cancer (18).

To devise an exceptional treatment plan for patients who have undergone left breast-conserving cancer surgery, we proposed the use of a half-field technique combined with VMAT and DIBH to ensure precise irradiation. To validate our protocol, four plans (free breathing (FB) IMRT (F-IMRT), DIBH IMRT (D-IMRT), F-VMAT, and D-VMAT) were designed, and the corresponding dosimetry parameters were compared to determine the better technique for patients undergoing radiotherapy for left breast cancer.

## 2 Data and methods

### 2.1 Patient datasets

Twenty-one patients with breast cancer treated at our institution from September 2022 to August 2023 underwent radiotherapy. The inclusion criteria were as follows: (1) remnant breast cancer following breast-conserving surgery and (2) a Karnofsky score (KPS) greater than 90. The exclusion criterion was the inability to achieve a breath-hold of more than 30 seconds following standard respiratory training. The study included 3 patients with TisN0M0, 16 patients with T1N0M0, and 2 patients with T2N0M0, all graded according to the 8th edition of the American Joint Committee on Cancer (AJCC) staging criteria for breast cancer. The patients ranged in age from 30 to 59 years, with a median age of 48 years. All patients signed informed consent to undergo radiotherapy and volunteered to participate in this study, which was approved by the Medical Ethics Committee of our institution.

### 2.2 Patient positioning and simulation

All patients were fixed with a vacuum pad (68 NL Coloredi) in the supine position with the ipsilateral arm raised above the top of the head and the head tilted sideways. After undergoing standard breathing training, patients were scanned using a CT simulation positioning machine (Brilliance Big Bore, Philips, USA) to obtain two sets of scans, one under the FB technique and the other under the DIBH technique. The scan range was up to the lower edge of the mandible to 10 cm below the breast skin fold (10th thoracic vertebra level), with a slice thickness of 5 mm. The reconstructed images were transmitted to an Eclipse 15.6 planning system (Varian Medical System, Palo Alto, USA).

### 2.3 Target volume delineation

The target volumes were outlined according to the guidelines for breast cancer developed by the American Radiation Therapy Oncology Group (RTOG), which defines the whole-breast clinical target volume (CTV) as all breast tissue on the affected side and the whole thoracic major muscle fascia but not the muscle tissues of the ribs or chest wall. The CTV was then uniformly expanded by 0.5 cm, and the anterior boundary was collected to 0.3 cm subcutaneously to obtain the planned target area (PTV). The organs at risk (OARs) mainly included the heart, left anterior descending (LAD) artery, left lung (lung_L), right lung, spinal cord, and contralateral mammary glands.

### 2.4 Treatment planning

**Figure 1:**
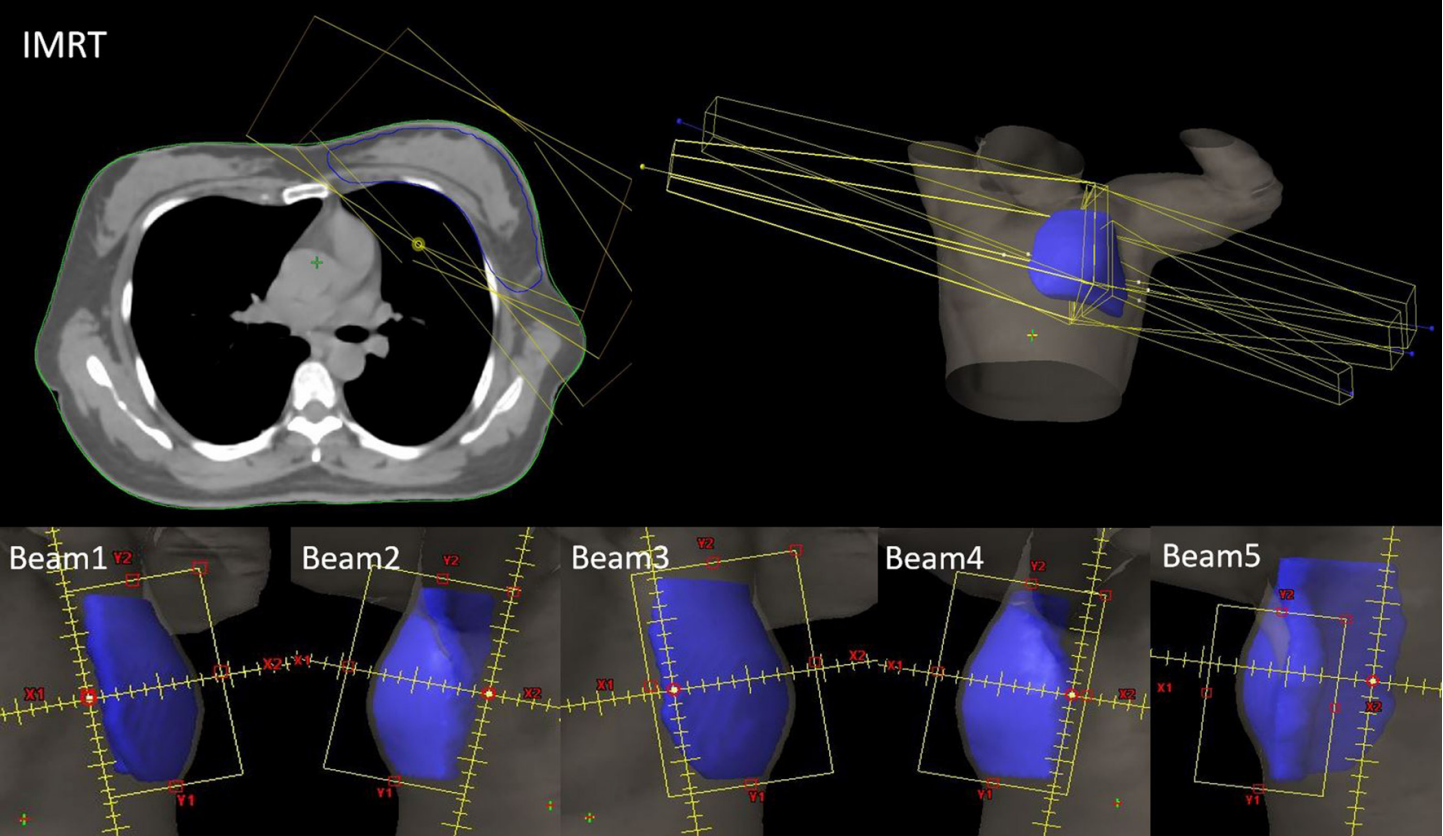
IMRT plan field setting and field beam’s eye view (BEV).

**Figure 2:**
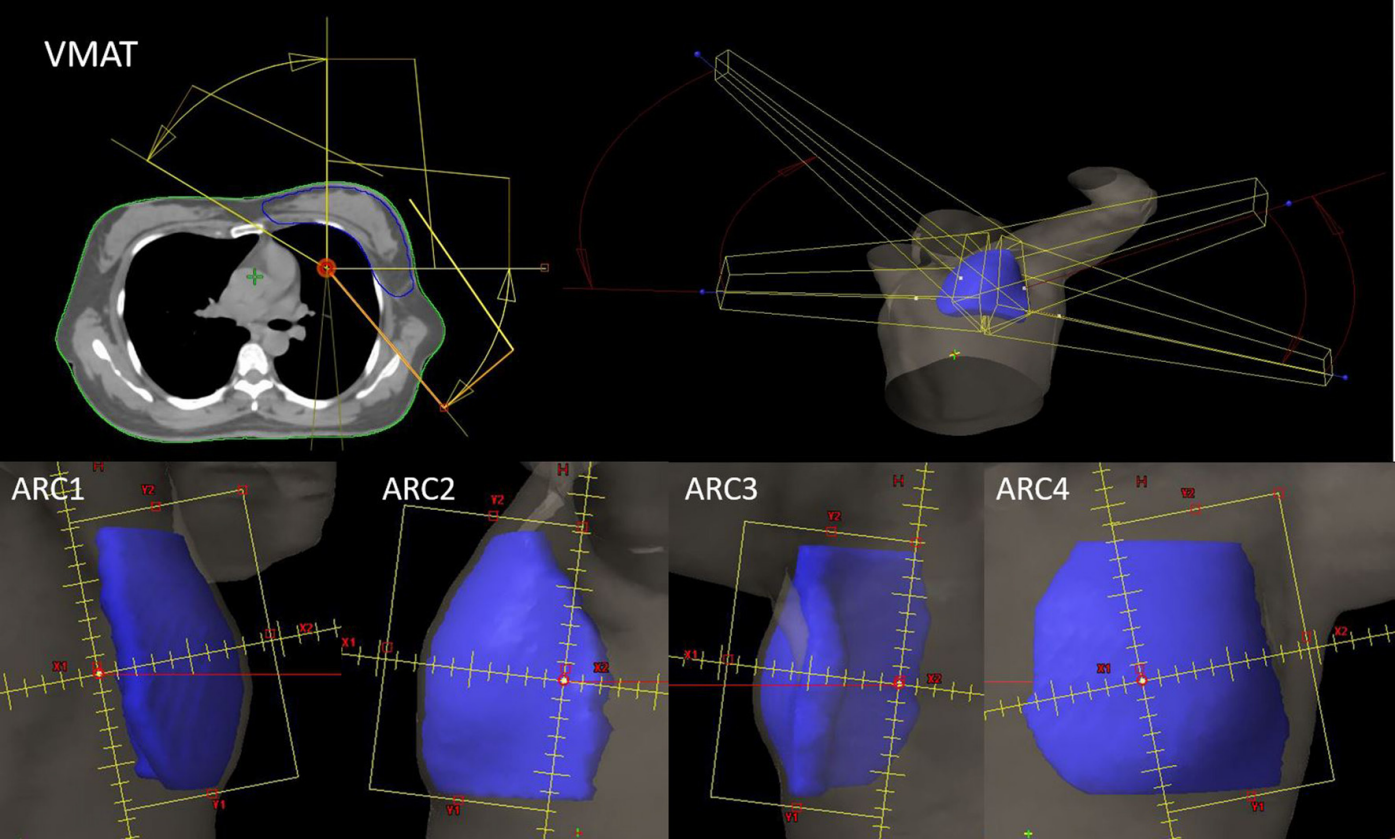
VMAT plan field setting and field BEV.

The Eclipse 15.6 planning system was used to design IMRT and VMAT plans under both the FB and DIBH techniques, producing the F-IMRT, F-VMAT, D-IMRT, and D-VMAT plans. In all plans, the doses to the PTV were 50 Gy, with 2.0 Gy per fraction per day, and 95% of the PTV was required to receive 100% of the prescribed dose. The dose limits for the OARs were as follows: lung_L V5 <50%, V20 <25%, V30<20%, heart Dmean<5 Gy, spinal cord Dmax <40 Gy, and contralateral breast V5 <1%, where Vx represents the percentage volume receiving a dose of x Gy. A VitalBeam linear accelerator was operated with 6 MV of X-ray energy, maintaining a dose rate of 600 motor units (MU)/min and utilizing a dose calculation grid of 0.25 cm. Progressive resolution optimization (PO, Photon Optimizer 15.6.06) was chosen as the inverse optimization algorithm, whereas the AXB algorithm (Acuros External Beam, Acuros XB 15.6.06) was employed for precise dose calculations. The IMRT plan involved use of the half-beam tangent strength tuning technique. As shown in **Figure 1**, the gantries of the two main tangent fields, Beam 1 and Beam 2, were positioned at 300° and 120°, respectively, and 3 auxiliary fields were set on their bases. The gantries of Beam 3, Beam 4 and Beam 5 were positioned at 315°, 105° and 145°, respectively; the partial lead door of Beam 5 was locked to reduce the volumes of the heart and axilla that were irradiated, and the collimators of all beams were adjusted to minimize the volume of lung_L that was irradiated. To reduce the dose effect of the type of respiratory exercise, the blade position was adjusted using the Skin Flash Tool in the Eclipse system. A total of 1 cm of the skin was exposed to the shooting field. The VMAT plans included four partial arcs with gantry angles of 300° -0°, 90° -145°, 145° -90°, and 0° -0° -300°, as shown in **Figure 2**. To achieve half-beam irradiation, collimator jaw X1 was closed and X2 was open for ARC 1 and ARC 2, while the opposite configuration was used for ARC 2 and ARC 3. Anterior expansion of the target area was considered for pseudotissue compensation.

### 2.5 Evaluation indicators

All four plans were normalized based on the prescription dose covering 95% of the PTV. The plans were evaluated using dose−volume histograms (DVHs) in accordance with the recommendations of the International Commission on Radiation Units and Measurements (ICRU) Report 83 (23). The evaluation metrics included the V95%, D98%, and D2% of the PTV, along with the conformity index (CI) and homogeneity index (HI), calculated as follows:


(1) $$\text{CI}=\;(V_{\text{PTV\&}50}/V_{\text{PTV}})*({\text{V}}_{\text{PTV\&}50}/{\text{V}}_{50})$$

(2) HI = (D2%−D98%)/D50%

**Figure 3:**
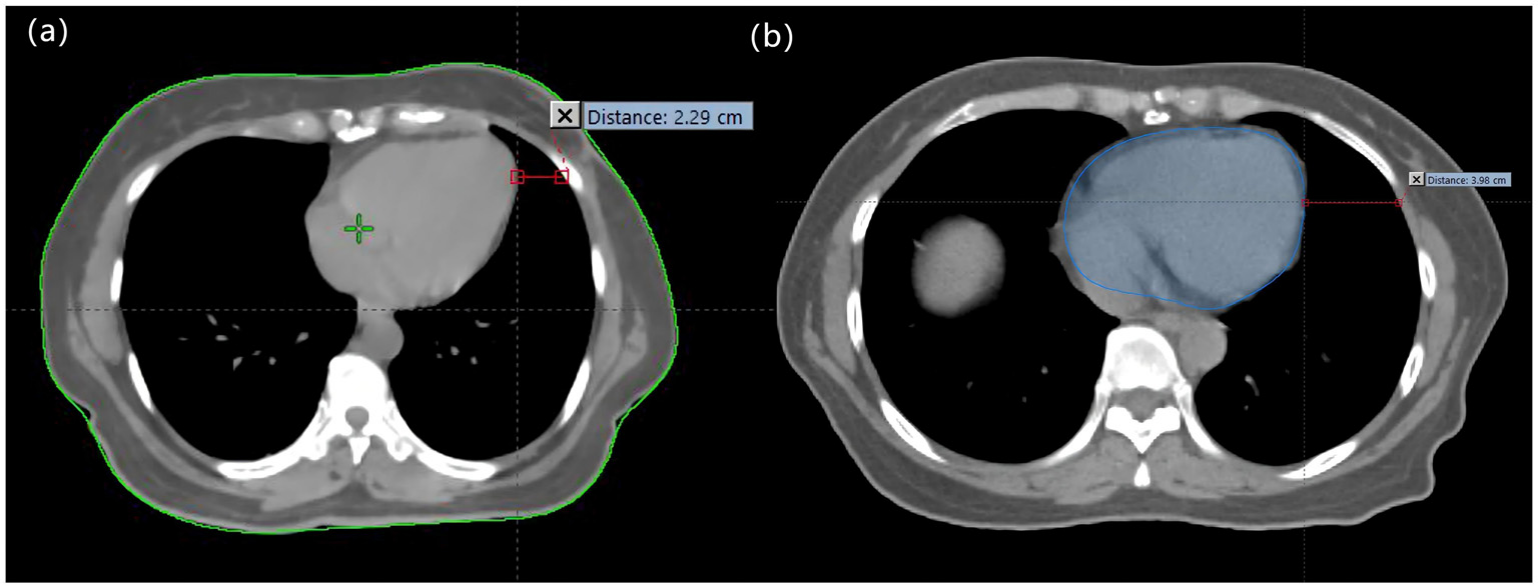
Measurement of the HCD on CT transverse imaging **(A)** and coronary imaging **(B)**.

V_50_ represents the volume of the body receiving 50 Gy, V_PTV_ represents the volume of the PTV, V_PTV50_ represented the volume within the PTV that received 50 Gy, D2% represents the dose delivered to 2% of the PTV, D98% represents the dose delivered to 98% of the PTV, and D50% represented the median dose of the PTV. The following parameters were calculated: V2.5, V5, V10, V20, V30, mean dose (Dmean), and left lung volume (LVL) for lung_L; V2.5, V5 and Dmean for lung_R; V5, V10, V20, D1%, Dmax, Dmean, and heart volume (HV) for the heart; Dmax and Dmean for the LAD artery; Dmax and Dmean for the contralateral breast; and the heart-to-chest distance (HCD) was defined as the horizontal distance between the most lateral margin of the heart and the chest wall (24), as depicted in **Figure 3**. Additionally, the following volume differences were calculated: ΔLVL, representing the difference in left lung volume between the DIBH and FB plans; ΔHV, indicating the difference in heart volume between the DIBH and FB plans; and ΔHCD, representing the difference in HCD between the DIBH and FB plans.

### 2.6 Statistical methods

SPSS 26.0 statistical analysis software was used to analyze the data. Variables are represented as ( $\bar{\text{x}}$ + s), and comparisons between groups were performed with the paired t test, Pearson correlation analysis was performed between pairs of variables; a correlation coefficient with an absolute value (|R|) between 0.8-1.0, 0.6-0.8 indicating a strong correlation, 0.4-0.6 indicating a moderate correlation, 0.2-0.4 indicating a weak correlation, and 0-0.2 indicating a very weak or no correlation. For all statistical tests, *p* < 0.05 represented statistical significance.

## 3 Results

### 3.1 Variations in the volumes of lung_L and the heart and in the HCD with the DIBH and FB techniques

Compared with those obtained with the FB technique, with the DIBH technique, the volume of the left lung was on average 837.6 cm^3^ greater, the heart volume was 33.2 cm^3^ lower, and the HCD was 1.3 cm greater; all differences were statistically significant (p <0.05), as shown in **Table 1**.

**Table 1:** Comparison of the volumes of and the distances between the left lung and heart calculated with the two breathing modes (DIBH and FB) (n=21, $\bar{x}$ + s).

Parameter	FB	DIBH	T	*p*
LVL(cm^3^)	1036.8 ± 199.9	1874.4 ± 300.9	-21.727	0.000
HV(cm^3^)	489.1 ± 84.1	455.9 ± 81.5	3.646	0.002
HCD(cm)	2.2 ± 0.8	3.5 ± 0.8	-11.146	0.000

### 3.2 Comparison of dosimetry parameters for PTV among the four plans

**Figure 4:**
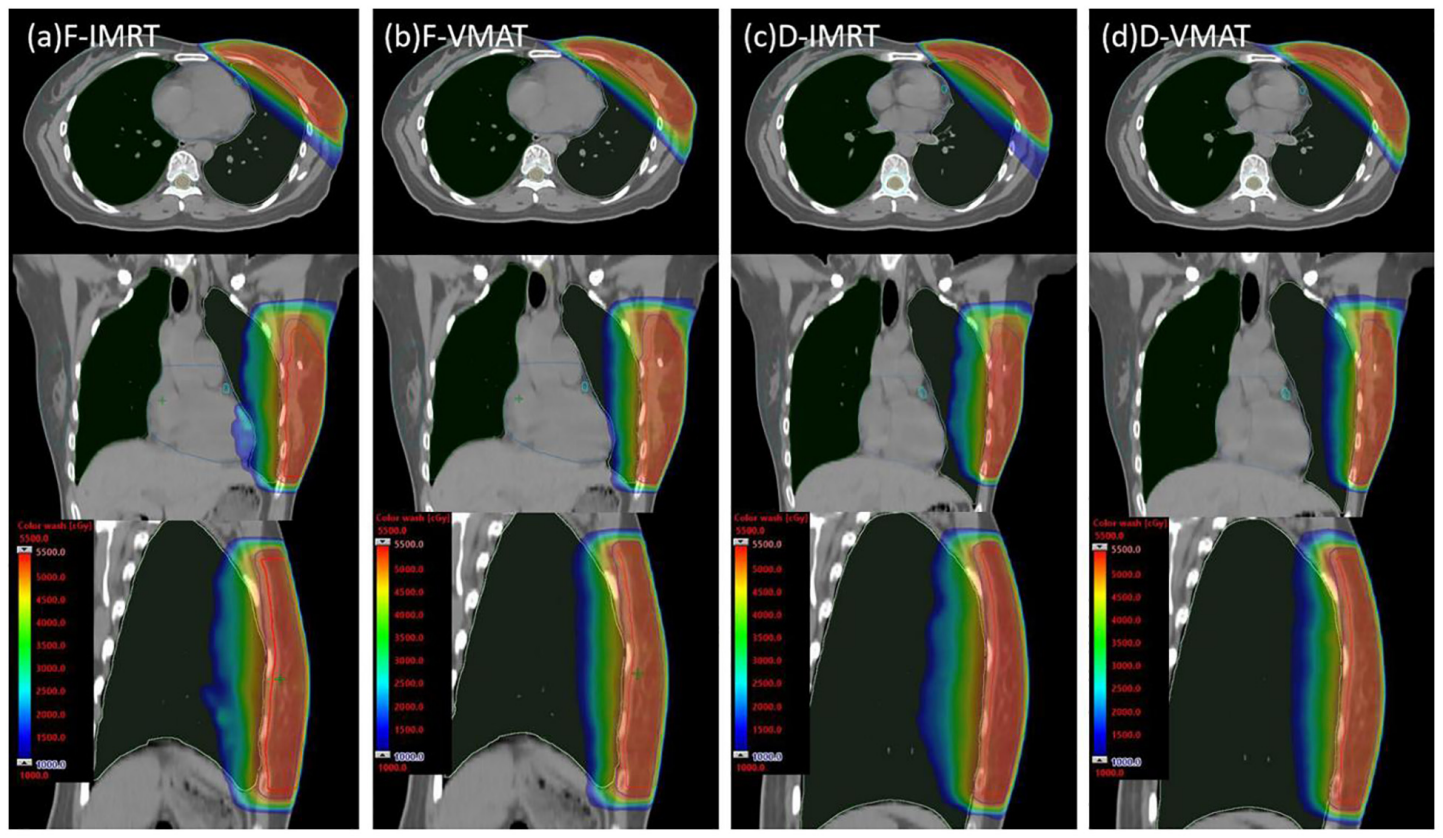
Dose distribution plots of the four plans in the transverse, coronal, and sagittal orientations (CTV contoured in red, PTV contoured in blue, heart contoured in deep sky blue, lung_L contoured in light green, lung_R contoured in dark green, LAD artery contoured in cyan, and breast_R contoured in sea green). **(A)** F-IMRT, **(B)** F-VMAT, **(C)** D-IMRT, **(D)** D-VMAT.

**Table 2** illustrates the doses delivered to the PTV across the four plans. Generally, the four plans demonstrated minor variations in PTV dosimetry parameters. Compared with the F-IMRT plan, the D-VMAT plan yielded a significantly greater D98% (p < 0.05). Additionally, compared to both the F-IMRT and D-IMRT plans, the D-VMAT plan exhibited a slightly elevated D2% (p < 0.05). However, when compared with the D-IMRT plan, the D-VMAT plan was marginally inferior with regard to the HI (p < 0.05). Furthermore, the average number of MUs for the D-VMAT plan was 433.7 ± 20.4, which was notably lower than the 734.1 ± 84.5 MUs for the F-IMRT plan and the 694.7 ± 62.6 MUs for the D-IMRT plan (p < 0.05). **Figure 4** shows the dose distributions of all four plans for the transverse, coronal, and sagittal views.

**Table 2:** Comparison of PTV dosimetry parameters obtained with the four plans for left breast radiotherapy (n=21, $\bar{x}$ + s).

Parameters	F-IMRT(1)	D-IMRT(2)	F-VMAT(3)	D-VMAT(4)	p -value		
					1vs4	2vs4	3vs4
V95%(%)	99.1 ± 0.3	99.2 ± 0.2	99.1 ± 0.2	99.2 ± 0.2	0.199	0.748	0.064
D98%(Gy)	48.7 ± 0.3	48.8 ± 0.2	48.8 ± 0.2	48.8 ± 0.1	0.048^*^	0.218	0.230
D2%(Gy)	53.8 ± 0.4	53.9 ± 0.4	54.2 ± 0.5	54.2 ± 0.5	0.000^**^	0.005^**^	0.340
CI	0.792 ± 0.034	0.797 ± 0.027	0.796 ± 0.025	0.798 ± 0.023	0.312	0.849	0.783
HI	0.098 ± 0.011	0.099 ± 0.010	0.103 ± 0.011	0.104 ± 0.010	0.051	0.022^*^	0.818
MU	734.1 ± 84.5	694.7 ± 62.6	429.4 ± 21.5	433.7 ± 20.4	0.000^**^	0.000^**^	0.472

The asterisk (*) indicated p < 0.05, and the double asterisk (**) indicated p < 0.01.

### 3.3 Comparison of dosimetry parameters for the OARs across the four plans

**Figure 5:**
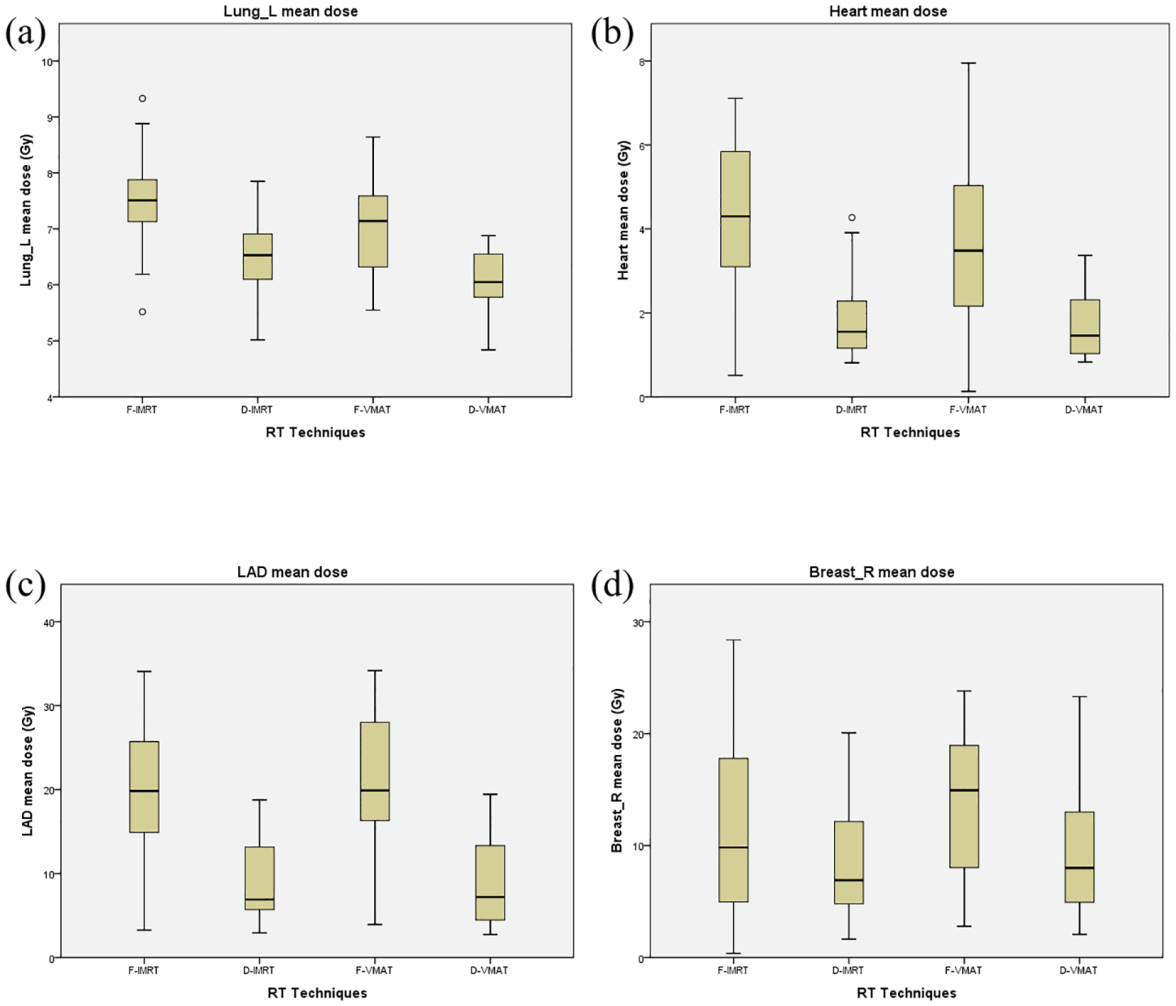
Comparison of the average dose to organs at risk for the F-IMRT, F-VMRT, D-IMRT and D-VMAT schedules in the four plans. **(A)** Lung_L, **(B)** Heart, **(C)** LAD, **(D)** Breast_R.

The doses received by the OARs in the four plans are shown in **Table 3**. Regarding lung_L, D-VMAT yielded a higher V2.5 than D-IMRT but a slightly lower value than F-VMAT (p < 0.05). D-VMAT also yielded a significantly lower lung_L V5 than both the F-IMRT and F-VMAT plans (p<0.05), but a comparable value to the D-IMRT plan (p>0.05). Furthermore, the D-VMAT plan demonstrated significantly lower lung_L V10, V20, V30, and Dmean values than did the F-IMRT, D-IMRT, and F-VMAT plans (p < 0.05). For the heart, the D-VMAT plan yielded significantly lower V5, V10, V20, and Dmean values for the heart than did F-IMRT, D-IMRT, and F-VMAT (p < 0.05). Moreover, the D1% and Dmax values of the heart with the D-VMAT plan were lower than those with both the F-IMRT and F-VMAT plans (p < 0.05). For the LAD artery, the Dmax and Dmean values obtained with D-VMAT were significantly lower than those obtained with the F-IMRT and F-VMAT plans (p < 0.05). The Dmax value for breast_R obtained with the D-VMAT plan was greater than that obtained with the D-IMRT plan but lower than that obtained with the F-VMAT plan (p <0.05). The Dmean of breast_R was lower when obtained with the D-VMAT plan than when obtained with the F-VMAT plan (p <0.05). The mean doses received by the left lung, heart, LAD artery, and right breast in each of the four treatment plans are graphically represented in **Figure 5**, providing a clear visualization of their distribution.

**Table 3:** Comparison of OAR dosimetry parameters among the four plans for left breast radiotherapy (n=21, $\bar{\text{x}}$ ± s).

Structure	Parameters	F-IMRT(1)	D-IMRT(2)	F-VMAT(3)	D-VMAT(4)	*p*-value		
						1vs4	2vs4	3vs4
Lung_L	V2.5(%)	36.8 ± 4.1	36.0 ± 4.1	40.6 ± 4.3	38.3 ± 3.7	0.372	0.000^**^	0.000^**^
	V5(%)	27.0 ± 2.9	25.1 ± 2.8	26.58 ± 3.0	24.8 ± 2.2	0.000^**^	0.405	0.002^**^
	V10(%)	20.3 ± 2.8	17.5 ± 2.9	18.3 ± 2.5	16.2 ± 2.2	0.000^**^	0.000^**^	0.001^**^
	V20(%)	13.7 ± 2.5	11.5 ± 2.2	12.0 ± 2.4	10.7 ± 2.0	0.000^**^	0.030^*^	0.001^**^
	V30(%)	8.8 ± 1.7	7.7 ± 1.2	7.9 ± 1.5	6.9 ± 1.1	0.000^**^	0.000^**^	0.005^**^
	Dmean(Gy)	7.5 ± 0.9	6.5 ± 0.7	7.0 ± 0.8	6.1 ± 0.5	0.000^**^	0.002^**^	0.000^**^
Lung_R	V2.5(%)	0.7 ± 1.0	0.4 ± 0.6	0.4 ± 0.7	0.2 ± 0.2	0.020^*^	0.349	0.187
	Dmean(Gy)	0.2 ± 0.1	0.1 ± 0.1	0.2 ± 0.1	0.1 ± 0.1	0.232	0.664	0.019^*^
Heart	V5(%)	18.1 ± 7.7	7.9 ± 5.2	14.4 ± 7.3	4.8 ± 3.6	0.000^**^	0.000^**^	0.000^**^
	V10(%)	11.8 ± 6.0	3.7 ± 3.8	9.4 ± 5.5	2.2 ± 2.4	0.000^**^	0.004^**^	0.000^**^
	V20(%)	7.2 ± 4.3	1.5 ± 2.3	5.9 ± 3.8	1.1 ± 1.4	0.000^**^	0.036^*^	0.000^**^
	D1%(Gy)	37.1 ± 12.7	16.6 ± 12.9	38.9 ± 12.8	16.6 ± 12.9	0.000^**^	0.474	0.000^**^
	Dmax(Gy)	48.0 ± 8.1	31.3 ± 11.6	48.9 ± 5.9	32.0 ± 14.5	0.000^**^	0.571	0.000^**^
	Dmean(Gy)	4.3 ± 2.0	1.9 ± 1.0	3.5 ± 2.2	1.7 ± 0.8	0.000^**^	0.007^**^	0.000^**^
LAD	Dmax(Gy)	44. ± 10.5	28.6 ± 10.5	46.1 ± 9.3	28.4 ± 14.5	0.000^**^	0.869	0.000^**^
	Dmean(Gy)	19.6 ± 8.1	9.2 ± 4.8	20.8 ± 8.2	9.0 ± 5.4	0.000^**^	0.621	0.000^**^
Breast_R	Dmax(Gy)	11.2 ± 7.8	8.2 ± 6.0	13.7 ± 6.8	9.8 ± 6.3	0.169	0.027^*^	0.026^*^
	Dmean(Gy)	0.8 ± 0.7	0.7 ± 0.6	1.5 ± 1.3	1.0 ± 0.9	0.324	0.160	0.003^*^

The asterisk (*) indicated p < 0.05, and the double asterisk (**) indicated p < 0.01.

### 3.4 Correlation analysis between ΔLVL and ΔHCD and dose difference parameters

**Figure 6:**
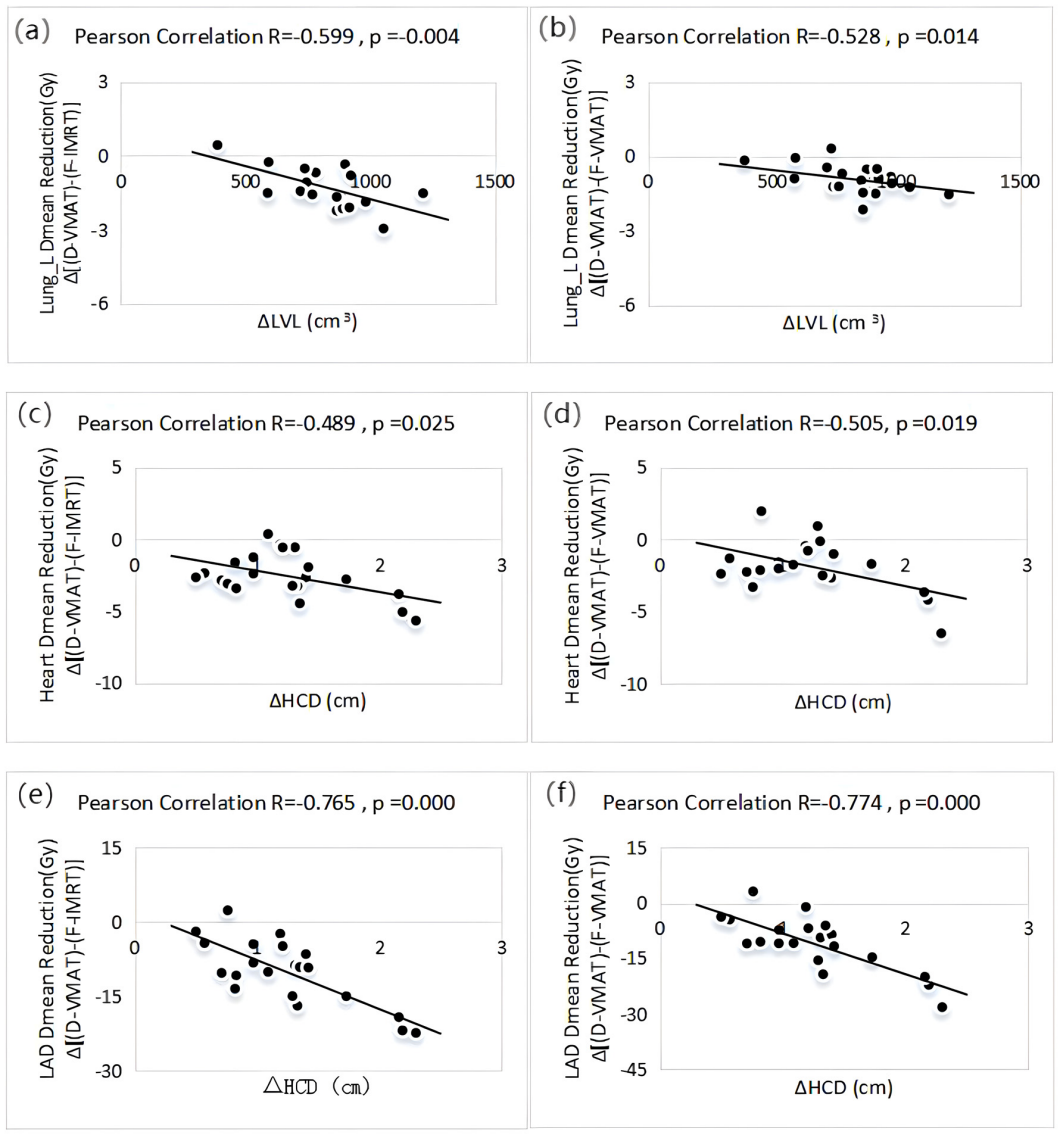
**(A)** Correlation between ΔLVL and ΔDmean of lung_L in the Plans D-VMAT and F-IMRT (R=-0.599, *p* =0.004). **(B)** Correlation between ΔLVL and ΔDmean of lung_L in the Plans D-VMAT and F-VMAT (R=-0.528, *p* =0.014). **(C)** Correlation between ΔHCD and ΔDmean of heart in the Plans D-VMAT and F-IMRT (R=-0.489, *p* =0.025). **(D)** Correlation between ΔHCD and ΔDmean of heart in the Plans D-VMAT and F-VMAT ( R=-0.505, *p* =0.019). **(E)** Correlation between ΔLVL and ΔDmean of LAD artery in the Plans D-VMAT and F-IMRT (R=-0.765, *p* =0.000). **(F)** Correlation between ΔLVL and ΔDmean of LAD artery in the Plans D-VMAT and F-VMAT (R=-0.774, *p* =0.000).

As depicted in **Figure 6**, the ΔLVL value was moderately negatively correlated with the ΔDmean value of lung_L calculated between the D-VMAT and F-IMRT plans and between the D-VMAT and F-VMAT plans (R = -0.599 and -0.528, respectively, *p* < 0.05). Similarly, a negative correlation was observed between the ΔHCD value and the ΔDmean value of the heart for the above two comparisons, with correlation coefficients of R = -0.489 and -0.505, respectively (*p* < 0.05). Additionally, ΔHCD was strongly negatively correlated with the ΔDmean value of the LAD artery calculated between the D-VMAT and F-IMRT plans and between the D-VMAT and F-VMAT plans (R = -0.765 and -0.774, respectively, *p* = 0.000).

## 4 Discussion

In this study, a comparative dosimetric analysis was conducted to evaluate the D-VMAT plan with respect to the F-IMRT, D-IMRT, and F-VMAT plans. Although no significant superiority was evident in the CI or HI for the PTV, the D-VMAT plan exhibited notable dosimetric advantages in safeguarding OARs. Specifically, the Dmean values of OARs such as the heart and lung_L were significantly lower in the D-VMAT plan than in the other three plans. Furthermore, the Dmax and Dmean values of the LAD were also lower in the D-VMAT plan than in the F-IMRT and F-VMAT plans. In summary, the D-VMAT plan demonstrated superior performance in terms of dosimetric benefits compared to the other three plans.

In this study, the IMRT plans employed two opposing half-beam tangential fields as the primary radiation sources, supplemented by three additional auxiliary fields, as depicted in **Figure 1**. Specifically, Beam 1 and Beam 2 served as the half-beam tangential fields, whereas Beam 3, Beam 4, and Beam 5 constituted the three auxiliary radiation fields. The half-beam tangent technique reduces scattering and low-dose volumes in breast cancer radiotherapy (18). As shown in **Figure 2**, for VMAT plans employing half-beam tangent rotating arc irradiation, the half-beam tangent arc adopts an independent jaw that can be moved to block half of the field along the central axis to eliminate beam divergence. During continuous rotating half-beam irradiation with ARC 1 and ARC 4, jaw X1 is turned off and jaw X2 is open, while ARC 2 and ARC 3 employ the opposite configuration. Lai Y et al. (25) demonstrated that the combination of a half-bundle with VMAT significantly reduced the radiation dose to the heart and affected lung of patients with left breast cancer with respect to IMRT. In this study, the D-VMAT plan was compared with the F-IMRT, D-IMRT and F-VMAT plans. In terms of HI, the D-VMAT plan yielded slightly higher values than did the F-IMRT and D-IMRT plans. However, there was no significant difference between the values obtained with the D-VMAT and F-IMRT plans, perhaps because of the small number of patients treated with the respective plans. Bi et al. (26) also concluded that the HI of VMAT was slightly greater than that of IMRT. Yu PC et al. (27) showed that the mean MU of a breast cancer VMAT plan was approximately 40% lower that obtained with an IMRT plan. This finding is similar to the results of this study, which revealed that the mean MU in the D-VMAT plan was 433.7, which was significantly lower than that in the F-IMRT (734.1) and D-IMRT plans (694.7) (*p <*0.05).

In this study, the DIBH method effectively increased the lung_L volume by an average of 837.6 cm³, thereby decreasing the density of radiation delivered to the lung. Correspondingly, the D-VMAT plan exhibited a marked reduction in the Vx (>5 Gy) and Dmean values of lung_L with respect to the D-IMRT plan (*p*<0.05). The V5 value of lung_L in the D-VMAT plan was similar to that in the D-IMRT plan, while the V2.5 value was significantly greater in the D-VMAT plan than in the D-IMRT plan (*p*<0.05). This observation indicates that the D-VMAT plan offers a substantial advantage in terms of dose distribution in high-dose areas (those receiving >5 Gy) of lung_L. Both plans were similar for 5 Gy, but the D-VMAT plan exposed larger low-dose areas (those receiving <5 Gy) than did the IMRT plan. However, previous research has indicated that VMAT plans tend to yield a significantly greater V5 for the lungs than IMRT plans (20). Specifically, Yu PC et al. (27) reported that the V5 of the left lung in a DIBH-VMAT plan was 31.7%, surpassing the 28.5% in a DIBH-IMRT plan. This difference could be explained by our use of the half-beam technique. It is crucial to note that, regarding the V2.5 value of lung_L, the D-VMAT plans consistently yielded greater values than the D-IMRT plans. Therefore, extra caution is advised when managing low-dose exposure to lung_L in the D-VMAT plan. Nonetheless, in terms of the average dose delivered to lung_L, the D-VMAT plan had a significantly lower dose than the other three plans considered in this study. Ultimately, these findings indicate that the D-VMAT plan offers superior protection for the left lung. Therefore, when evaluating overall performance, the D-VMAT plan is a superior option for protecting lung_L.

In left-sided breast cancer RT plans, the heart and LAD artery are the most important organs at risk, requiring large weights in the target function. Studies have shown that the exposed dose to the heart is still relatively high with both IMRT and VMAT schedules, with an average doses > 7.8 Gy, even up to 15.2 Gy (28–31). The DIBH technique is one of the most popular methods used in breast cancer radiotherapy plans (13, 32), as it can increase the distance between the heart and the chest wall while ensuring the optimal dose in the target area, thus effectively reducing the dose to the heart (9, 33, 34). In this study, the V5, V10, V20, D1%, Dmax and Dmean values of the heart in the F-IMRT and F-VMAT plans were significantly greater than those in the D-VMAT plan, and the Dmax and Dmean of the LAD artery in the F-IMRT and F-VMAT plans were significantly greater than those in the D-VMAT, aligning with the findings of previous studies (27, 35). In this study, the cardiothoracic distance was 1.3 cm greater with the DIBH technique than with the FB technique, creating space for a dose drop outside the target area, further protecting the heart and LAD artery. This finding indicates that the D-VMAT plan significantly outperforms the F-IMRT and F-VMAT plan in the protection of the heart and its substructures. In this study, the D-VMAT V5, V10, V20, and Dmean values were significantly lower than those of the D-IMRT program (p <0.05). For the Dmax and Dmean in the right breast, the VMAT plans yielded slightly greater values than did the IMRT plan, similar to the findings of Yu et al. (27). Because the VMAT technique results in greater dose scattering on the contralateral mammary gland, special attention should be given to the exposed dose of the healthy breast when choosing the D-VMAT plan.

Mohamad et al. (36) studied 22 left breast cancer patients and showed that the difference in the maximum cardiac distance between FB and DIBH plans was significantly associated with the decrease in the mean cardiac dose caused by the DIBH plan. In this study, a moderate correlation was observed between the mean dose reduction in the left lung and the left lung volume difference (ΔLVL) when comparing the F-VMAT to the D-VMAT plan. Specifically, the patients’ ΔLVL was 768.8 cm^3^, while the left lung ΔDmean decreased 0.91 Gy from the F-VMAT to the D-VMAT plan; in one patient, ΔLVL was 917.1 cm^3^, and their left lung ΔDmean decreased by 1.52 Gy from the F-VMAT to the D-VMAT plan. Furthermore, the heart mean dose difference and LAD artery mean dose difference were similarly correlated with the ΔHCD. The results of these correlation analyses suggest that the greater the ΔLVL and ΔHCD are in the DIBH respiratory mode, the greater the reduction in the dose delivered to the left lung, heart, and LAD artery.

## 5 Conclusion

For patients who underwent radiotherapy following breast-conserving surgery for left breast cancer, the D-VMAT plan exhibited dosimetry parameters in the target PTV comparable to those of the F-IMRT, D-IMRT, and F-VMAT plans. When considering OARs, the D-VMAT plan offered enhanced protection for those such as the left lung, heart, and LAD artery. Nevertheless, compared to the D-IMRT plan, the D-VMAT plan may result in a minor elevation in the V2.5 value for lung_L and the Dmax value for the heart. The integration of the DIBH respiratory mode with half-beam VMAT technology in the D-VMAT plan offers patients a superior dosimetry distribution, thus implying important clinical implications.

## Data Availability

The original contributions presented in the study are included in the article/supplementary material. Further inquiries can be directed to the corresponding author.
